# Modulation of Cerebrospinal Fluid Dysregulation via a SPAK and OSR1 Targeted Framework Nucleic Acid in Hydrocephalus

**DOI:** 10.1002/advs.202306622

**Published:** 2024-02-14

**Authors:** Qiguang Wang, Jian Cheng, Fei Liu, Jianwei Zhu, Yue Li, Yuxuan Zhao, Xiang Li, Huan Zhang, Yan Ju, Lu Ma, Xuhui Hui, Yunfeng Lin

**Affiliations:** ^1^ Department of Neurosurgery West China Hospital Sichuan University Chengdu 610041 P.R. China; ^2^ Institutes for Systems Genetics Frontiers Science Center for Disease‐Related Molecular Network West China Hospital Sichuan University Chengdu 610041 P.R. China; ^3^ Department of Neurosurgery Sichuan Provincial People's Hospital University of Electronic Science and Technology of China Chengdu 610000 P.R. China; ^4^ Core facilities West China Hospital Sichuan University Chengdu 610041 P.R. China; ^5^ State Key Laboratory of Oral Diseases National Center for Stomatology National Clinical Research Center for Oral Diseases West China Hospital of Stomatology Sichuan University Chengdu 610041 P. R. China; ^6^ Sichuan Provincial Engineering Research Center of Oral Biomaterials Sichuan University Chengdu 610041 P.R. China; ^7^ National Center for Translational Medicine Shanghai Jiao Tong University Shanghai 200240 P.R. China

**Keywords:** cerebrospinal fluid hypersecretion, choroid plexus, cytokines, DNA nanomaterials, hydrocephalus, macrophages

## Abstract

Hydrocephalus is one of the most common brain disorders and a life‐long incurable condition. An empirical “one‐size‐fits‐all” approach of cerebrospinal fluid (CSF) shunting remains the mainstay of hydrocephalus treatment and effective pharmacotherapy options are currently lacking. Macrophage‐mediated ChP inflammation and CSF hypersecretion have recently been identified as a significant discovery in the pathogenesis of hydrocephalus. In this study, a pioneering DNA nano‐drug (TSOs) is developed by modifying S2 ssDNA and S4 ssDNA with SPAK ASO and OSR1 ASO in tetrahedral framework nucleic acids (tFNAs) and synthesis via a one‐pot annealing procedure. This construct can significantly knockdown the expression of SPAK and OSR1, along with their downstream ion channel proteins in ChP epithelial cells, thereby leading to a decrease in CSF secretion. Moreover, these findings indicate that TSOs effectively inhibit the M0 to M1 phenotypic switch of ChP macrophages via the MAPK pathways, thus mitigating the cytokine storm. In in vivo post‐hemorrhagic hydrocephalus (PHH) models, TSOs significantly reduce CSF secretion rates, alleviate ChP inflammation, and prevent the onset of hydrocephalus. These compelling results highlight the potential of TSOs as a promising therapeutic option for managing hydrocephalus, with significant applications in the future.

## Graphical abstract

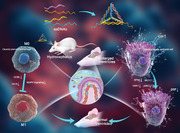

## 1 Introduction

Hydrocephalus is one of the most common neurosurgical disorders worldwide and a life‐long incurable condition. The prevalence of infantile hydrocephalus is ≈1 case per 1000 births,^[^ ^1^ ^]^ and accounts for nearly 29.4% of pediatric neurosurgical admissions.^[^ ^2^ ^]^The elevated intracranial pressure in hydrocephalus always damages the periventricular white matter, impairing brain development in children and causing neurodegeneration in adults.^[^ ^3^ ^]^ In severe cases, intracranial hypertension can lead to acute brainstem herniation and death.^[^ ^4^ ^]^ Surgical cerebrospinal fluid (CSF) shunting, an empirical “one‐size‐fits‐all” approach remains the mainstay of hydrocephalus treatment.^[^ ^5^ ^]^ However, shunting failure, caused by obstruction or infection, affects 40% of patients. Furthermore, around 60% of surgically treated patients experience lifelong neurologic symptoms, such as motor skills impairment, cognitive deficits, or epilepsy.^[^ ^6^, ^7^ ^]^ Therefore, there is an urgent need to develop targeted pharmacotherapeutic strategies for patients with hydrocephalus.

It is widely acknowledged that hydrocephalus arises from CSF accumulation due to disrupted CSF circulation homeostasis.^[^ ^8^ ^]^ However, there are no appropriate fast‐acting, low‐risk targeted pharmacotherapeutic strategies for treating hydrocephalus. Recently, Kahle et al. made a significant discovery, elucidating that choroid plexus (ChP) inflammation due to intraventricular hemorrhage (IVH) or infection leads to CSF hypersecretion, thereby promoting the development of hydrocephalus.^[^ ^9^ ^]^ The integrated multi‐omics study depicted CNS border‐associated macrophages (BAMs) on the apical ChP membrane always serve as the “‘first responders”’ to microorganisms in the CSF.^[^ ^10^ ^]^ The activated macrophages trigger CSF “cytokine storm” and engage their corresponding receptors on ChP epithelial cells, thus inciting the inflammatory response.^[^ ^10^ ^]^ Inflammation in epithelial cells leads to an upregulation of phosphorylation SPAK/OSR1–NKCC1 at the choroid plexus apical membrane, resulting in CSF hypersecretion,^[^ ^9^, ^10^ ^]^ and Karimy et al. found that genetic depletion of SPAK normalized CSF hypersecretion rates and reduced PHH symptoms.^[^ ^9^ ^]^ Furthermore, SPAK/OSR1 plays a regulatory role in multiple ion transport proteins associated with CSF secretion, such as ATP1a1, KCNJ13, and CLIC6.^[^ ^10^ ^]^ SPAK and OSR1 are important upstream regulators of NKCC1/2 and NCC.^[^ ^11^ ^]^ In addition to its involvement in CSF hypersecretion, SPAK/OSR1‐NKCC1 cascade has been implicated in regulating immune response, and inhibiting SPAK‐NKCC1 cascades has shown potential in reducing inflammation response and modulating macrophage activation.^[^ ^12^, ^13^, ^14^, ^15^ ^]^ Hence, we aimed to design a new therapeutic strategy of both genetic inhibition of SPAK/OSR1 kinase in ChP epithelial cells and simultaneous inhibition of ChP macrophage activation to treat hydrocephalus.

RNA interference using antisense oligonucleotides (ASOs) is a naturally occurring gene downregulation mechanism.^[^ ^16^ ^]^ ASOs, artificially synthesized single‐stranded nucleic acids ranging from 18 to 25 bases, offer a unique capability to target nascent pre‐mRNAs within the nucleus and mRNAs in the cytoplasm.^[^ ^16^ ^]^ However, the clinical application of ASOs remains constrained due to the limited cellular penetration and vulnerability to degradation. Hence, multiple delivery systems have been explored by researchers worldwide. Tetrahedral framework nucleic acids (tFNAs), formed by self‐assembled four‐single‐stranded DNAs (ssDNA) through highly specific Watson−Crick base pairing, have recently garnered significant attention in biomedical fields.^[^ ^17^, ^18^ ^]^ They have several advantages, including straightforward synthesis, good biocompatibility, ease of editing, and efficient entry into mammalian cells.^[^ ^19^ ^]^ Moreover, tFNAs exhibit remarkable anti‐oxidation and anti‐inflammatory properties by modulating macrophage responses.^[^ ^20^ ^]^ Given these exceptional properties, tFNAs emerge as a highly effective vehicle in the management of hydrocephalus.

**Scheme 1:**
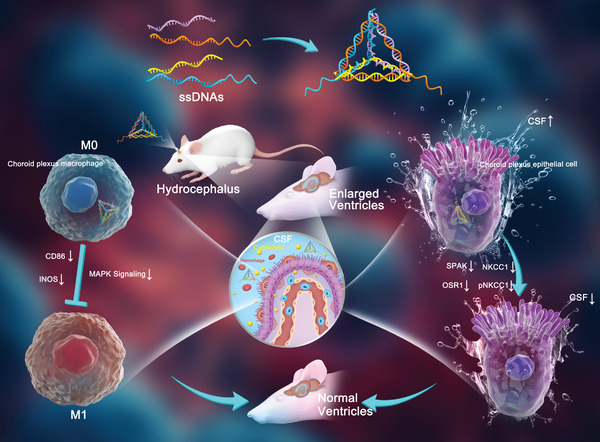
TSOs inhibit CSF hypersecretion by downregulating SPAK and OSR1 in ChP epithelial cells and inhibiting the phenotypic switch of ChP macrophages from M0 to M1 through MAPK signaling pathways, thus preventing hydrocephalus formation.

In this study, we present a pioneering DNA nano‐drug, termed tFNA‐SPAK@OSR1ASOs (TSOs), which are designed to attach both SPAK ASO and OSR1 ASO to tFNAs. We modified S2 ssDNA with SPAK ASO and S4 ssDNA with OSR1 ASO, and synthesized the four ssDNAs using a single‐step annealing method, thereby achieving higher synthesis efficiency and greater stability. Mechanistically, TSOs reduce the expression of SPAK, OSR1, NKCC1, and pNKCC1 in ChP epithelial cells, thereby leading to a decrease in CSF secretion. Remarkably, TSOs inhibit the phenotypic switch of ChP macrophages from M0 to M1 and attenuate the inflammatory response. In a rat hydrocephalus model, we have observed that TSOs significantly decrease CSF secretion, reduce ventricle size, mitigate choroid plexus macrophage infiltration, and diminish the levels of proinflammatory cytokines in the CSF. This work pioneers a novel application by combining two distinct functional ASOs with tFNAs and could have potential clinical applications in treating hydrocephalus in the future (**Scheme** **1**).

## 2 Results and Discussion

### 2.1 Synthesis and Characterization of tFNA‐SPAK@OSR1ASOs (TSOs)

**Figure 1:**
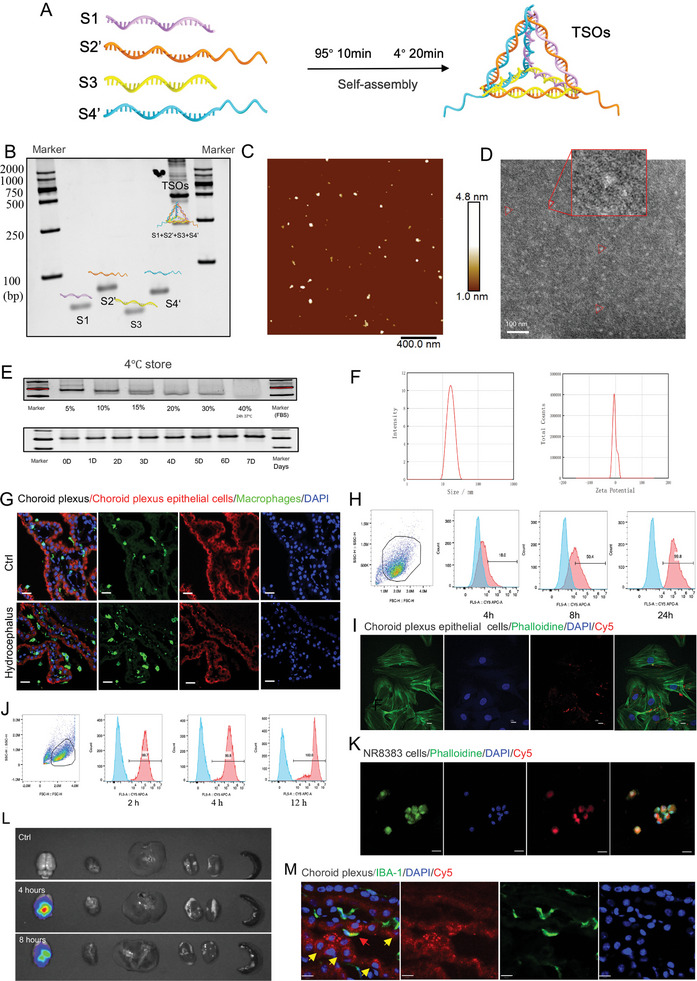
Synthesis, characteristics, and uptake of TSOs. A) Schematic diagram of TSO fabrication. B) 8% PAGE verified the successful synthesis of TSOs. C) AFM images of the molecular structure of TSOs. Scale bars: 400 nm. D) TEM images of the molecular structure of TSOs. Scale bar: 100 nm. E) Serum and storage stability of TSOs. F) The zeta potentials and hydrodynamic sizes of TSO measured by DLS. G) Immunofluorescence showing the increased IBA1+ macrophage accumulation at the surface of choroid plexus epithelial cells in rat PHH models. H) Flow cytometry to see the uptake of TSOs in primary choroid plexus epithelial cells. I) The uptake of TSOs by primary choroid plexus epithelial cells at 6 h. Scale bar: 10 µm. J) Flow cytometry to assess the uptake of TSOs in rat macrophages NR8383 cells. K) The uptake of TSOs in NR8383 cells. Scale bar: 20 µm. L) After intraventricular injection, in vivo imaging showed the majority of TSOs remained within the cerebrospinal fluid distribution region for at least 8 h. M) Cy5 signal intensities, upon intraventricular injection, were observed in IBA1‐positive epiplexus macrophages (red arrow) and choroid plexus epithelial cells (yellow arrow).

Tetrahedral DNA nanostructure had four single DNA strands and each ssDNA had 63 bases.^[^ ^21^ ^]^ In our study, we undertook structural modifications of tFNAs to function as an effective vehicle for facilitating the intracellular transport of antisense oligonucleotides (ASOs). Specifically, we modified S2 ssDNA with SPAK ASO and S4 ssDNA with OSR1 ASO (Table S1, Supporting Information). These modified TSOs were synthesized using a one‐pot annealing procedure of heating at 95 °C for 10 min and then quickly cooling to 4 °C for 20 min (**Figure** **1**A). The successful self‐assembly of TSOs was confirmed by 8% polyacrylamide gel electrophoresis (Figure 1B and Figure S1A, Supporting Information). The morphology of TSOs was characterized by AFM (Figure 1C) and TEM (Figure 1D), and TSOs were a negatively charged triangle‐like structure with an approximate size of 17.42 nm (Figure 1D,F). We conducted an evaluation of the structural stability of TSOs and observed TSOs remained stable in 10% FBS for at least 24 h, this extended stability duration rendered TSOs highly suitable for most in vitro cell experiments (Figure 1E). They could be stable at 4 °C for at least 7 days (Figure 1E). The zeta potential of TSOs was −3.118 mV (Figure 1F). Furthermore, stability analysis showed that TSOs are relatively stable in the range of pH 7–9, but in NaCl and CaCL_2_ solutions, the stability is relatively poor (Figure S1B,C, Supporting Information). To achieve a more efficient synthesis of TSOs, the system of 100 µL and pH 8.0 of TM buffer are necessary, and keep in mind ssDNA should be stored at −20 °C and keep the synthetic process at 4 °C.

The accumulation of ChP macrophages and crosstalk between macrophages and ChP epithelial cells play a pivotal role in CSF hypersecretion and the development of hydrocephalus (Figure 1G). Hence, we investigated the biosafety of TSOs in these two cell types. Primary rat ChP epithelial cells and rat macrophages (NR8383) were treated with different concentrations of TSOs, and a CCK8 kit was used to assess their cytotoxicity. We found that the cells treated with 300 nM TSOs showed decreased cell viability than the concentration of 250 nM (Figure S1D, Supporting Information). Hence, we determined a concentration of 250 nM as the optimal concentration in the subsequent cell experiments.

Subsequently, we assessed the uptake of TSOs in primary ChP epithelial cells and rat macrophages (NR8383) using flow cytometry techniques and immunofluorescence. Flow cytometry analysis revealed that 50.4% of choroid plexus epithelial cells had internalized TSO after 8 h, which dramatically increased to 99.8% at 24 h (Figure 1H). Similarly, NR8383 cells displayed a significant uptake of 99.7% within 2 h of incubation (Figure 1J). The immunofluorescence images confirmed the distribution of TSOs within the cytoplasm of both cell types (Figure 1I,K). Following the intraventricular injection of TSOs, in vivo imaging analysis revealed that the majority of TSOs remained within the cerebrospinal fluid distribution region, exhibiting a robust fluorescence signal for at least 8 h (Figure 1L). In vitro investigations demonstrated a significant uptake of TSOs by NR8383 cells at 2 h and by ChP epithelial cells at 8 h, affirming the efficacy of ventricle route administration for TSOs. Notably, in PHH ChP, macrophages coexist amidst the ChP epithelial cells and represent a minor population. The Cy5 signal intensities, upon intraventricular injection, were observed in IBA1‐positive epiplexus macrophages (Figure 1M, red arrow) and choroid plexus epithelial cells (Figure 1M, yellow arrow). These results provided valuable insights into the efficient cellular uptake of TSOs, thus laying the foundation for their potential therapeutic applications in managing hydrocephalus.

### 2.2 Role of TSOs in Knockdown SPAK and OSR1 in Choroid Plexus Epithelial Cells

The vectorial ion transport at the choroid plexus epithelium secrets>500 cc per day CSF into the brain ventricular spaces.^[^ ^22^ ^]^ CSF secretion requires the coordinated function of multiple ion and water transport proteins. The CCCs (cation‐chloride cotransporters) family, composed of Na+, K+‐coupled CL‐importers (NCC, NKCC1, and NKCC2), and the K+‐coupled CL‐ exporters (KCC1‐4), have been found to play a central role in regulating CSF secretion.^[^ ^9^, ^23^ ^]^ The vectorial ion transport NKCC1 and KCC1 at the choroid plexus epithelium cell, contributes more than 50% of CSF production.^[^ ^23^ ^]^ More importantly, the Ste20‐type Ser–Thr protein kinases SPAK (SPS1‐related proline/alanine‐rich kinase) and OSR1 (oxidative stress‐responsive kinase 1) are considered master regulators of the CCCs.^[^ ^24^ ^]^ Besides, several ion channel proteins such as CLIC6, KCNJ13, and ATP1A1 are also closely related to the CSF secretion in hydrocephalus and are regulated by SPAK/OSR1.^[^ ^10^ ^]^ The upregulated pSPAK/OSR1‐NKCC1, resulting from ChP inflammation, leads to a notable 3.5‐fold increase in CSF secretion and significantly exacerbates the occurrence of hydrocephalus. Karimy et al. reported that genetic depletion of SPAK can normalize CSF hypersecretion rates, as does treatment with drugs targeting the SPAK‐NKCC1 complex.^[^ ^9^ ^]^ Consequently, it is hypothesized that inhibiting SPAK/OSR1 in epithelial cells may effectively mitigate hydrocephalus by reducing CSF secretion.

**Figure 2:**
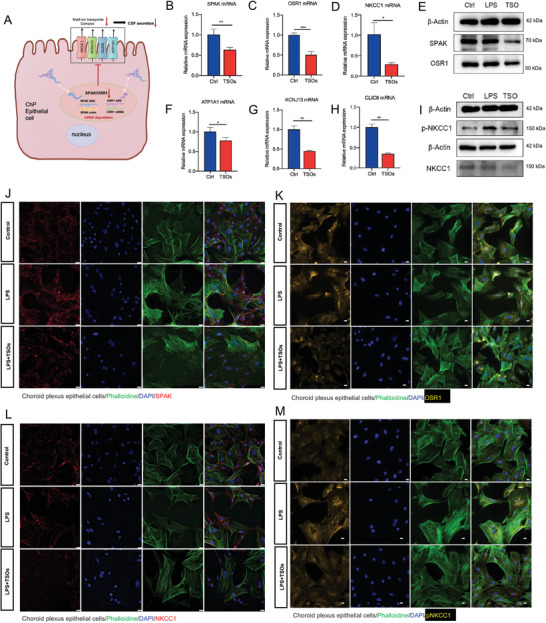
TSOs significantly downregulate the expression of SPAK, OSR1, NKCC1, and pNKCC1 in primary choroid plexus epithelial cells. A) Schematic illustration of the function of TSOs in primary choroid plexus epithelial cells. B–D) The mRNA expression level of B) SPAK, C) OSR1, and D) their downstream NKCC1 in primary ChP epithelial cells after TSOs treatment. E) Western blot results showing changes of SPAK and OSR1, after TSOs treatment. F–H) QPCR showing TSOs downregulate the downstream ion channel related to CSF secretion F) ATP1A1, G) KCNJ13, and H) CLIC6, data are presented as mean ± SD (*n* = 3). *p < 0.05, **p < 0.01, ***p < 0.001. I) Western blot results showing changes in NKCC1 and pNKCC1 expression levels after TSO treatment. J–M) Immunofluorescence images showed the J) SPAK, K) OSR1, L) NKCC1, and M) pNKCC1 expression reduced after TSO treatment. Scale bar: 20 µm.

Several strategies targeting the ATP‐binding site of SPAK/OSR1 have been put forward. The introduction of STOCK1S‐50699 suppressed in vitro phosphorylation of SPAK/OSR1 and NKCC1,^[^ ^25^ ^]^ but in vivo pharmacokinetics were found to be unfavorable.^[^ ^26^ ^]^ Closantel, a livestock antiparasitic drug, has emerged as the first candidate for in vivo pharmacological SPAK inhibition,^[^ ^27^ ^]^ but its use in humans is contraindicated due to retinal toxicity.^[^ ^28^ ^]^ Zhang et al. designed a novel SPAK inhibitor that can inhibit NKCC1 and stimulate KCCs by decreasing their SPAK‐dependent phosphorylation.^[^ ^26^ ^]^ Here, we designed and synthesized a new SPAK/OSR1 inhibitor by attaching SPAK ASO and OSR1 ASO to the tFNAs. We then investigated the efficacy of our newly designed nanomaterial, TSOs, in downregulating the expression of SPAK and OSR1 in primary ChP epithelial cells (**Figure** **2**A). After incubation with TSOs for 24 h, QPCR analysis revealed a significant reduction of SPAK and OSR1 mRNA expression in ChP epithelial cells (Figure 2B,C). Building upon recent multi‐omics research on hydrocephalus, SPAK/OSR1 regulated not only NKCC1 expression but also the expression of CLIC6, KCNJ13, and ATP1A1, which are all associated with CSF hypersecretion.^[^ ^10^ ^]^ Therefore, we further investigated the expression of downstream genes of SPAK/OSR1 after TSO treatment. The results demonstrated that TSOs effectively reduced mRNA transcription levels of NKCC1, CLIC6, KCNJ13, and ATP1A1 (Figure 2D,F**–**H).

The above‐mentioned study discovered that TSOs could effectively downregulate SPAK and OSR1 levels, as well as their downstream genes at the mRNA levels. To further validate these findings, we conducted experiments to assess the impact of TSOs on SPAK and OSR1 expression at the protein level. ChP epithelial cells were treated with TSOs for 48 h; subsequently, Western blot and immunofluorescence methods were utilized to analyze the protein levels. The results demonstrated a notable downregulation in the expression of both SPAK and OSR1 proteins (Figure 2E,J,K). The protein of pNKCC1 represents the Na+/K+/2CL‐ cotransporter (NKCC1), a significant ion channel responsible for nearly half of CSF production.^[^ ^23^, ^29^ ^]^ The expression of NKCC1 is tightly regulated by SPAK and/or OSR1 through a regulatory mechanism.^[^ ^30^, ^31^ ^]^ Therefore, we explored whether TSOs could effectively downregulate the expression of both NKCC1 and pNKCC1 proteins, thus evaluating the ability of TSOs to modulate the downstream protein expression of SPAK/OSR1. The results showed that TSOs significantly downregulated NKCC1 and pNKCC1 (Figure 2I,L,M). Additionally, we investigated whether simultaneous knockdown of both SPAK and OSR1 would result in a synergistic effect on their downstream protein, NKCC1. The findings demonstrated that TSOs exhibited a more pronounced knockdown effect on NKCC1 expression, in both protein and mRNA levels, compared to tFNA‐SPAKASOs (Figure S1E,F, Supporting Information).

Based on our findings, TSOs demonstrate remarkable efficacy in suppressing the expression of SPAK and OSR1 in ChP epithelial cells. Moreover, they play a pivotal role in significantly downregulating various downstream ion channels, including pNKCC1, NKCC1, CLIC6, KCNJ13, and ATP1A1. These findings suggest the potential of TSOs in modulating crucial targets involved in CSF hypersecretion, providing valuable insights for hydrocephalus management.

### 2.3 TSOs Blocked Transition of Macrophages to M1‐Like Phenotype through Inhibiting MAPK Signaling Pathway

Recently, Kahle et al. unveiled a novel mechanism in hydrocephalus that crosstalk between macrophages and ChP epithelial cells drives CSF hypersecretion.^[^ ^10^ ^]^ IVH or intraventricular infection triggers the accumulation of peripheral monocytes at the choroid plexus, subsequently leading to the activation of ChP macrophages. These activated macrophages, in turn, initiate a CSF “cytokine storm” by releasing various inflammatory cytokines into the CSF.^[^ ^10^ ^]^ The cytokines can act on the ChP epithelial cells via their corresponding receptors and trigger an upregulated phosphorylation of SPAK/OSR1–NKCC1 at the choroid plexus apical membrane, thereby inducing CSF hypersecretion.^[^ ^10^, ^32^ ^]^ Given this newfound understanding, pharmacotherapy aimed at targeting the inflammatory ChP macrophages may present a promising and innovative treatment avenue for hydrocephalus.

Tetrahedral framework nucleic acids (tFNAs) represent a promising DNA nanomaterial. The tFNAs are self‐assembled by four‐single‐stranded DNAs (ssDNA) and they have stable structures and show excellent biological effects in various diseases.^[^ ^19^ ^]^ Notably, tFNAs exhibit exceptional internal tunability, cellular uptake, and biocompatibility, alongside good stability and low toxicity.^[^ ^33^ ^]^ Our previous studies found the tFNAs have anti‐inflammatory and antioxidant effects,^[^ ^34^ ^]^ effectively downregulating the expression of TNF‐α, IL‐1β, and IL‐6 in LPS‐induced macrophages.^[^ ^20^ ^]^ These properties position tFNAs as an ideal carrier to treat hydrocephalus. Furthermore, studies have unveiled the pivotal role of the SPAK‐NKCC1 cascade in modulating macrophage activation and proinflammatory cytokine production.^[^ ^14^, ^15^ ^]^ Specifically, overexpression of SPAK has been linked to increased proinflammatory cytokine production, while SPAK knockout has shown the potential to reduce inflammation response and mitigate inflammatory damage.^[^ ^12^, ^13^ ^]^ Genetic knockdown or pharmacological modulation of NKCC1 has demonstrated efficacy in alleviating the inflammation cascade in the lungs and inhibiting macrophage activation.^[^ ^14^, ^35^, ^36^ ^]^

**Figure 3:**
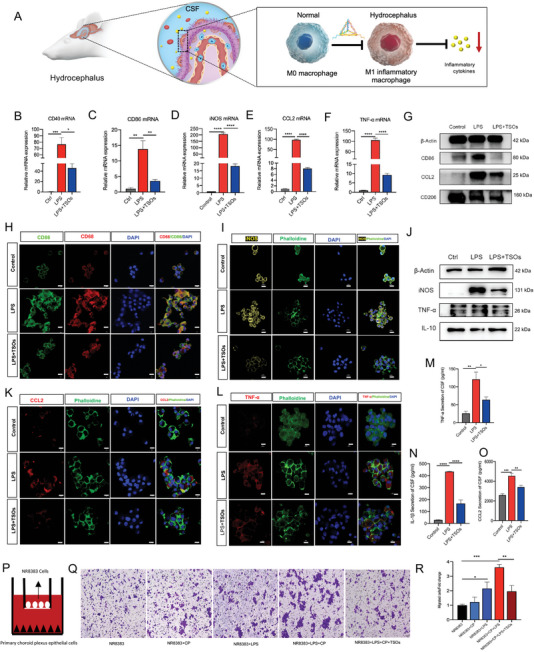
TSOs inhibit the M0 to M1 phenotypic switch of macrophages and suppress the inflammatory cytokines. A) Schematic diagram of TSOs inhibiting M0 to M1 phenotypic switch of macrophages. QPCR showing TSOs downregulated B) CD40, C) CD86, D) iNOS, E) CCL2, and F) TNF‐α, *p < 0.05, **p < 0.01, ***p < 0.001, ****p < 0.0001. G) Western blot detecting the expressions of CD86, CCL2, and CD206. H) Expression of CD86 under IF microscopy. I) IF staining of iNOS in NR8383 after LPS and TSO treatment. J) Western blot detecting the expressions of iNOS, pNF‐κB, TNF‐α, and IL‐10. K,L) Expression of K) CCL2 and L) TNF‐α after LPS and TSO treatment under IF microscopy. M–O) ELISA detecting the content of M) TNF‐α, N) IL‐1β, and O) CCL2 in NR8383 culture, *p < 0.05, **p < 0.01, ***p < 0.001, ****p < 0.0001. P) Schematic diagram of 2D‐transwell experiment. Q) Images of transwell see the vertical migrating ability of NR8383 in the co‐culture system at 24 h. R) The migration of NR8383 was quantitatively analyzed, *p < 0.05, **p < 0.01, ***p < 0.001. Data are presented as mean ± SD (*n* = 3).

The polarization and reprogramming of macrophages play a pivotal role in their functional versatility. In response to diverse microenvironments, primary macrophages (M0) can undergo polarization, adopting either pro‐inflammatory (M1) or anti‐inflammatory (M2) phenotypes.^[^ ^37^ ^]^ Notably, M1 macrophages express key markers such as leukocyte differentiation antigen (CD86) and nitric oxide synthase (iNOS), thereby producing inflammatory mediators including proinflammatory cytokines (e.g., tumor necrosis factor α [TNF‐α], interleukin‐6 [IL‐6]) and chemokine (C–C motif) ligand 2 (CCL2).^[^ ^38^ ^]^ In hydrocephalus, inflammatory stimuli such as heme and microorganisms, drive the polarization of choroid plexus macrophages from the M0 state to an M1‐like phenotype.^[^ ^39^, ^40^ ^]^ The activated macrophages release inflammatory cytokines, which interact with ChP epithelial cells through ligand‐receptor pairing, thus leading to heightened epithelial transport and subsequent CSF hypersecretion.^[^ ^10^ ^]^ Here, to elucidate the role of TSOs in ChP macrophage polarization, we utilized an LPS (1 µg ml^−1^)‐induced NR8383 cell model to simulate the activation of M1 macrophages in vitro. We then used QPCR (**Figure** **3**B–F), Western blot (Figure 3G,J), immunofluorescence (Figure 3H,I,K,L), and ELISA (Figure 3M–O) to find out the role of TSOs on macrophage polarization. The results demonstrated that TSOs significantly downregulated expression of iNOS and CD86, and concurrently inhibited the secretion of TNF‐a, IL‐1β, and chemokine CCL2 in LPS‐treated NR8383 cells. However, the expression of M2 markers such as IL‐10 and CD206 showed no significant change in LPS and TSO‐treated groups. Hence, we concluded that TSO administration blocked the transition of NR8383 macrophages from M0 to M1 phenotype and reduced the inflammation state, as evidenced by the downregulation of iNOS, CD86, and also multiple proinflammatory cytokines (Figure 3A).

The accumulation of macrophages at the ChP and crosstalk between macrophages and epithelial cells are key drivers of hydrocephalus.^[^ ^10^ ^]^ If the accumulation of ChP macrophages is suppressed, the hydrocephalus can be relieved.^[^ ^10^ ^]^ To investigate whether TSOs have the ability to regulate macrophage accumulation, we used a 2D transwell co‐culture system in vitro in the current study (Figure 3P). We cultured NR8383 cells in the upper chamber and primary ChP epithelial cells were placed in the lower chamber, after treating the lower chamber with LPS and TSOs, the number of macrophages migrated from the upper chamber to the lower chamber significantly decreased (Figure 3Q,R). Previous studies have highlighted the vital role of the CCL2 signaling molecule in recruiting macrophages to the ChP‐CSF interfaces.^[^ ^41^ ^]^ We then investigated whether TSO inhibited the release of CCL2 in LPS‐treated ChP epithelial cells. We measured the expression of CCL2 by ChP epithelial cells through QPCR, Western Blot, and Immunofluorescence (Figure S2A–C, Supporting Information). The results showed that TSOs weakened the ability of ChP epithelial cells to recruit macrophages by inhibiting CCL2 signaling.

**Figure 4:**
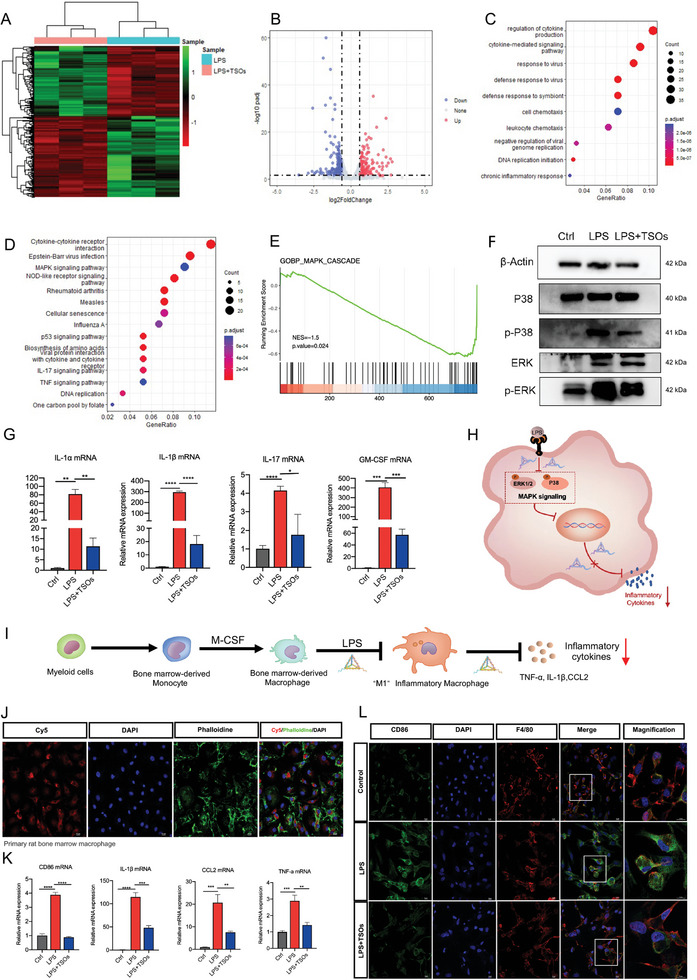
RNA‐sequencing reveals that TSOs inhibit macrophage polarization through the MAPK signaling pathway. A) Heat map showing the differentially expressed genes in the NR8383 cells treated with LPS and TSOs. B) Volcano plots showing DEGs. C) GO biological process analysis of differentially expressed genes. D) The KEGG pathways analysis showing the differentially expressed genes were primarily associated with the cytokine‐cytokine receptor interaction and MAPK signaling pathway. E) GSEA showed a significant decrease of MAPK pathways gene enriched. F) Western blot showing the protein levels of P38, p‐P38, ERK, and p‐ERK. G) QPCR showing that TSOs inhibited various proinflammatory cytokines in NR8383 cells, *p < 0.05, **p < 0.01, ***p < 0.001, ****p < 0.0001. H) Schematic diagram of the mechanism of TSOs in inhibiting macrophage polarization. I) The potential effect of TSOs on BM‐derived macrophage polarization upon LPS stimulation. J) The uptake of TSOs by BM‐derived macrophages at 6 h. Scale bar: 10 µm. K) QPCR showing TSOs downregulated CD86, IL‐1β, CCL2, and TNF‐α in BM‐derived macrophages,**p < 0.01, ***p < 0.001, ****p < 0.0001. F) IF staining of CD86 and F4/80 in BM‐derived macrophages after LPS and TSO treatments. Scale bar: 10 µm. Data are presented as mean ± SD (*n* = 3).

To gain deep insights into the molecular mechanism by which TSO inhibited NR8383 macrophage polarization from M0 to M1, we conducted RNA sequencing analysis. We performed statistical analysis on genes that were statistically and expressed by more than | log_2_foldchange |>0.6 and presented them in the form of heatmaps and volcano plots (**Figure** **4**A,B), with 203 genes being significantly down‐regulated and 176 genes being significantly up‐regulated. These differentially expressed genes were found to be enriched in functions related to the regulation of cytokine production and cytokine‐mediated signaling pathway (Figure 4C). Additionally, Kyoto Encyclopedia of Genes and Genomes (KEGG) analysis showed that the addition of TSOs affected cytokine‐cytokine receptor interaction, MAPK signaling pathway, NOD‐like receptor signaling, TNF signaling pathway, and IL‐17 signaling pathways (Figure 4D,E). MAPK signaling is associated with macrophage polarization, which can depress the expression of M1 macrophage‐specific markers and play roles in pro‐inflammatory pathways in macrophages.^[^ ^42^ ^]^ We then conducted Western blotting to assess the activations of MAPK subfamilies, including p38 and ERK1/2 and observed a significant downregulation following treatment with TSOs (Figure 4F). Additionally, QPCR analysis was performed to examine cytokine expression and showed downregulation of cytokines upon TSO application (Figure 4G). Mitogen‐activated protein kinase (MAPK) signaling, encompassing p38, ERK1/2, and JNK subfamilies, plays a pivotal role in regulating proinflammatory cytokines and the upregulation of multiple inflammation‐related genes.^[^ ^43^, ^44^ ^]^ Prior studies have demonstrated that ERK and p38 could increase the expression of cytokines such as TNF‐α and IL‐1β, along with macrophage polarization.^[^ ^45^ ^]^ p38α has important roles in activating feedback pathways that downregulate inflammation.^[^ ^46^ ^]^ Besides, several recent studies have found that MAPK signaling may play a significant role in kaolin‐induced hydrocephalus based on the high‐throughput RNA‐seq.^[^ ^47^ ^]^ Yue et al. reported that TGF‐β1 could induce fibrosis of MMCs via the p38 MAPK signaling pathway in hydrocephalus and can serve as a novel potential target for intervention.^[^ ^48^ ^]^ Hence, we concluded that TSO inhibited macrophage polarization from M0 to M1 and suppressed inflammation by downregulating the MAPK signaling pathway (Figure 4H).

To further explore whether TSOs can inhibit the differentiation of macrophages towards a pro‐inflammatory phenotype in the hydrocephalus microenvironment. We isolated myeloid cells from SD rat tibia and differentiation induction into bone marrow‐derived macrophages with M‐CSF (20 ng ml^−1^) (Figure 4I). Initially, we confirmed that TSOs can be taken in by the BM‐derived macrophages (Figure 4J). The expression of M1 biomarker CD86 in BM‐derived macrophages was detected by QPCR and immunofluorescence. After LPS activation, the expression of CD86 in BM‐derived macrophages significantly increased, and TSO treatment marginally decreased CD86 expression (Figure 4K,L). Furthermore, the administration of TSO significantly attenuated the pro‐inflammatory responses in LPS‐treated BM‐derived macrophages, as evidenced by reduced levels of TNF‐α, CCL2, and IL‐1β (Figure 4K and Figure S2, Supporting Information).

### 2.4 TSOs Inhibited the Occurrence and Progression of Hydrocephalus after IVH

**Figure 5:**
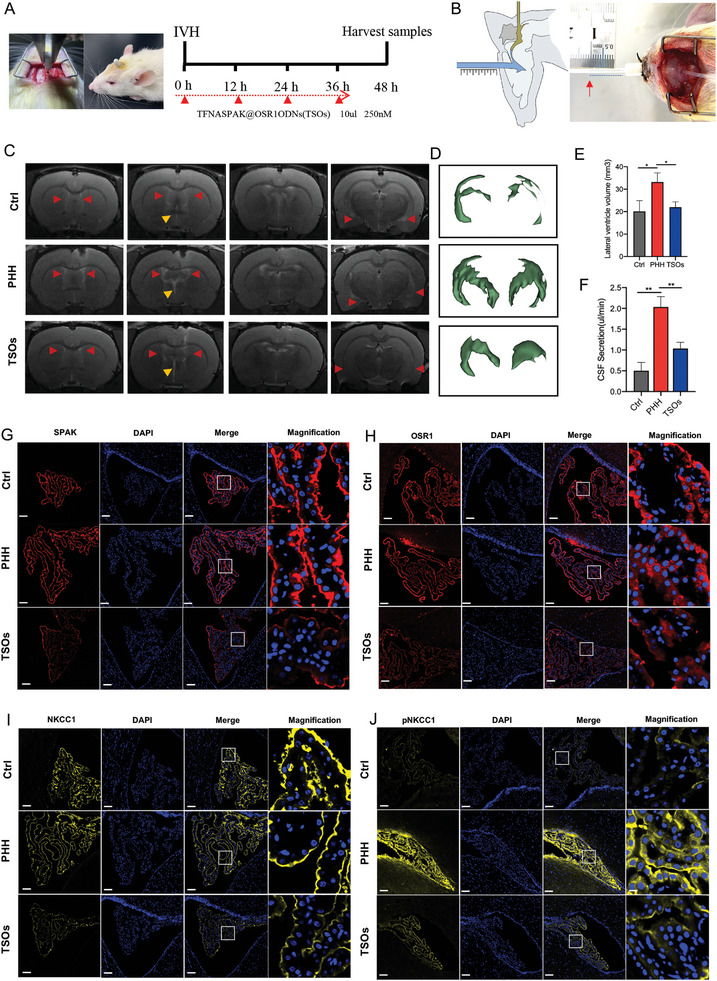
TSOs inhibit the occurrence of hydrocephalus after IVH. A) Illustration of drug delivery method and Schematic display of time nodes for drug injection in vivo. B) The methods of measuring CSF secretion rate. C) Represent 7‐T magnetic resonance images of Ctrl, PHH, and TSOs groups at 48 h. D) The 3D reconstructed images of lateral ventricles. E) Quantification volumes of the lateral ventricle based on the related T2‐weighted images (*n* = 3 animals per condition), *p < 0.05. F) Quantification of CSF secretion rates (*n* = 3 animals per condition), **p < 0.01. G–J) IF staining images of G) SPAK, H) OSR1, I) NKCC1, and J) pNKCC1 at the choroid plexus in Ctrl, PHH, and TSO treatment groups. Scale bar: 100 µm, entire images and scale bar: 10 µm, the magnified images.

To investigate the potential of TSOs in inhibiting hydrocephalus formation after IVH in vivo. We established a rat PHH model. Although tFNA possesses numerous advantages, including simple synthesis steps, stable structure, and efficient transport of therapeutic cargo into the cell, our previous studies have shown that a significant proportion of tFNAs get absorbed by the kidney and liver and metabolically eliminated after the tail vein injection.^[^ ^49^ ^]^ Inspired by the clinical practice of Ommaya drainage and ventricular drainage for treating hydrocephalus, we devised an intraventricular drug delivery device. This device was surgically implanted into the right lateral ventricle of rats and securely affixed on the skull (**Figure** **5**A), as previously detailed by our research team.^[^ ^50^ ^]^ Before injection into the rat ventricles, the TSO drugs were purified utilizing the Millipore Amicon ultrafiltration device (30 KDa) and subsequently redissolved with artificial cerebrospinal fluid. A comprehensive description of the purification method can be found in our previous study.^[^ ^21^ ^]^ Because the intraventricular drug injection has less impact on other organs, we investigated the potential toxic effects of TSOs on the heart, liver, lung, kidney, and spleen through the tail vein infection, and the results showed no significant toxic effects. The organs showed normal cell morphology, with no obvious inflammatory cell infiltration (Figure S2F, Supporting Information).

We then tested the impact of TSOs on the CSF secretion rate and CSF volume in vivo. The rate of CSF secretion was quantified using the method proposed by Karimy and his colleagues^[^ ^9^ ^]^ (Figure 5B) and CSF volume was evaluated through 7T small animal magnetic resonance imaging. Additionally, we utilized live T2‐weighted magnetic resonance (MRI) to quantify lateral ventricle volume. The results clearly demonstrated that treatment with TSO significantly reduced the volume of the lateral (red arrows) and the third (yellow arrows) ventricles (Figure 5C–E). Furthermore, TSOs significantly reduced the CSF secretion rate triggered by IVH (Figure 5F).

The secretion of CSF requires the coordinated function of multiple water and ion transport proteins.^[^ ^22^ ^]^ WNK‐SPAK/OSR1 kinases play integral roles in the regulation of intracellular Na+, K+, and Cl– homeostasis as well as cell volume homeostasis.^[^ ^51^, ^52^ ^]^ SPAK and OSR1 share high amino acid sequence homology in both their N‐terminal catalytic domain (96%), and their C‐terminal regulatory domain (67%).^[^ ^51^ ^]^ SPAK/OSR1 regulates multiple ion transport proteins, such as NKCC1, ATP1a1, KCNJ13, and CLIC6, which are essential to PHH pathophysiology in ChP epithelial cells; among them, NKCC1 is the most significant contributor, accounting for nearly half of CSF secretion.^[^ ^10^, ^23^ ^]^ IVH or intraventricular infection can significantly increase the levels of functional SPAK/OSR1‐NKCC1 and lead to CSF hypersecretion.^[^ ^9^ ^]^

We next investigated the in vivo knockdown efficiency of TSOs targeting the SPAK, OSR1, and their downstream NKCC1 and pNKCC1. In vivo experiments were conducted to investigate the mRNA levels of SPAK, OSR1, and their downstream ion channels. Following a 48‐h injection into the brain ventricles, QPCR analysis revealed TSO significantly downregulated the expression of SPAK, OSR1, NKCC1, ATP1A1, KCNJ13, and CLIC6 (Figure S3A, Supporting Information). We then investigated the effects of TSOs on SPAK/OSR1 at the protein level in PHH models. Besides, we focused on two crucial downstream molecules of SPAK/OSR, NKCC1 and its phosphorylated form (pNKCC1). Our results demonstrated that TSOs significantly suppressed the expression of these proteins in the choroid plexus (Figure 5G–J). Therefore, the above results depict TSOs have a promising effect in inhibiting the occurrence of hydrocephalus after IVH.

**Figure 6:**
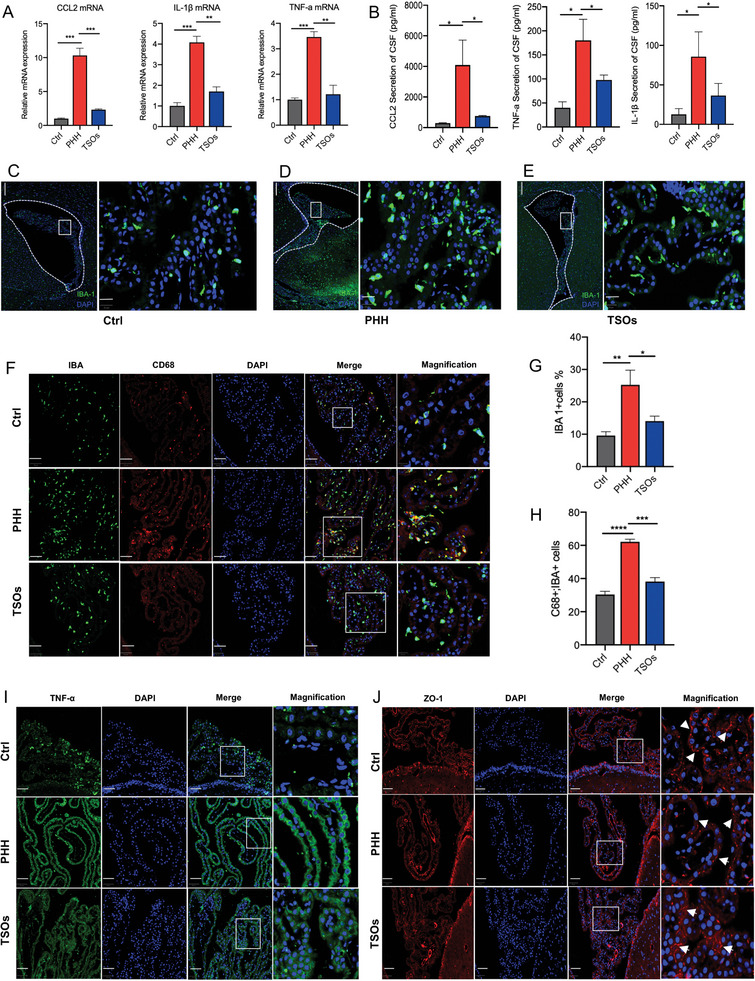
TSOs significantly inhibit ChP macrophage infiltration, ChP inflammation, and restore the blood‐CSF barrier function in PHH. A) QPCR showing that TSOs inhibited TNF‐α, IL‐1β, and CCL2 mRNA expression in choroid plexus (*n* = 3 animals per condition), **p < 0.01, ***p < 0.001. B) ELISA detected the content of CCL2, TNF‐α, and IL‐1β in CSF of Ctrl, PHH, and TSOs treated rats (*n* = 3 animals per condition), *p < 0.05. C) The number of IBA1^+^ macrophages in the choroid plexus of normal rats. D) Increased macrophage infiltration at the choroid plexus in PHH rats. E) The macrophage infiltration in PHH rats treated with TSOs. Scale bar: 200 µm, entire images and scale bar: 20 µm, the magnified images. F) The presence of CD68^+^IBA1^+^ macrophages in choroid plexus in Ctrl, PHH, and TSOs treated groups. Scale bar (50 µm, entire images and scale bar: 20 µm, the magnified images. G) The number of IBA1^+^macrophages in choroid plexus was quantitatively analyzed (*n* = 5 animals per condition), *p < 0.05, **p < 0.01. H) The number of CD68^+^IBA1^+^macrophages in the choroid plexus was quantitatively analyzed (*n* = 5 animals per condition), ***p < 0.001 and ****p < 0.0001. I) Expression of TNF‐α in Ctrl and PHH rats, and PHH rats treated with TSOs. Scale bar: 50 µm, entire images and scale bar: 20 µm, the magnified images. J) The expression of ZO‐1 in different groups. Scale bar: 50 µm, entire images and scale bar: 10 µm, the magnified images.

The CSF cytokines storm due to IVH can engage their cognate receptors on ChP epithelial cells and result in CSF hypersecretion.^[^ ^8^ ^]^ Given the ability of TSOs to regulate cytokines production in in vitro experiments. We then proceed to investigate the role of TSOs in modulating ChP inflammation in in vivo PHH models. The expression of TNF‐α, IL‐1β, and CCL2 in the choroid plexus was determined by QPCR (**Figure** **6**A) and immunofluorescence (Figure 6I and Figure S3B, Supporting Information). The cytokines secretion in CSF was measured by enzyme‐linked immunosorbent assay (ELISA) (Figure 6B). We observed increased levels of proinflammation cytokines including CCL2, IL‐1β, and TNF‐α in PHH models, indicating that IVH induced a proinflammatory state in the choroid plexus and CSF. After administration of TSOs, we found that TSOs significantly relieved ChP inflammation and reduced inflammatory cytokines in CSF. These findings demonstrate the potential of TSOs in inhibiting ChP inflammation. Besides, CSF CCL2 significantly decreased after TSO treatment. Recent studies have demonstrated that ChP inflammation is mediated by macrophages and macrophages are the first responders to microorganisms in the CSF^[^ ^10^ ^]^ and CCL2 signaling plays a central role in recruiting macrophages to the ChP‐CSF interfaces.^[^ ^53^, ^54^ ^]^ We subsequently explored the potential of TSOs to modulate macrophage infiltration in vivo, employing IBA‐1 staining, a pivotal biomarker for macrophages. We found TSO effectively impeded the infiltration of macrophages across the ChP barrier (Figure 6C–E,G). In fixed tissues, CD68 is exploited as a marker of increased macrophage activity.^[^ ^41^, ^55^ ^]^ In rats with PHH, a notable increase of CD68+ macrophages was observed in the choroid plexus. Treatment with TSOs resulted in a reduction in the accumulation of CD68+ macrophages (Figure 6F,H), suggesting their beneficial impact on inhibiting ChP macrophage activation.

The choroid plexus is formed by epithelial cells and surrounds a core of fenestrated capillaries.^[^ ^19^ ^]^ The choroid plexus epithelial cells are interconnected by TJ at the basement membrane, forming the blood‐CSF barrier.^[^ ^56^ ^]^ The functional tight junctions in ChP serve as not only a gatekeeper regulating peripheral toxins and nutrients but also the entry of peripheral immune cells.^[^ ^41^ ^]^ Previous studies have revealed that inflammation can disrupt the integrity of ChP barriers, leading to the accumulation of immune cells.^[^ ^57^, ^58^, ^59^ ^]^ In this study, we employed ZO‐1, a classical tight junction protein, as an indicator of blood‐CSF barrier integrity in PHH. The results revealed a disrupted ZO‐1 staining pattern in the choroid plexus of PHH. TSO treatment suppressed the break of ChP barrier integrity, revealed by increased expression of ZO‐1 proteins. Previous studies have reported proinflammatory cytokines such as IL‐12, IL‐1β, and tumor necrosis factor (TNF‐a) can block the epithelial barrier by downregulating the tight junction protein.^[^ ^13^ ^]^ Besides, the activation of SPAK‐NKCC1 is also associated with loss of blood–CSF barrier integrity.^[^ ^60^ ^]^ In the present study, we found that TSO treatment inhibited the ChP barrier integrity damage induced by PHH (Figure 6J). This beneficial effect of TSOs on ChP barrier function is likely attributed to its ability to alleviate ChP inflammation. Repurposing drugs that regulate ChP's immune‐secretory function may offer a non‐surgical treatment strategy for hydrocephalus.^[^ ^10^ ^]^ Such a new nano‐drug that targets choroid plexus neuroinflammation and CSF hypersecretion may prevent shunt dependence and reduce the lifelong morbidity and economic burden associated with hydrocephalus in the future.

## 3 Conclusion

The “cytokine storm” mediated by ChP macrophages and the upregulated phosphorylation of SPAK/OSR1 in choroid plexus epithelial cells constitute critical mechanisms in hydrocephalus. In the present study, we designed novel TDN‐based nanomaterials (TSOs) by attaching SPAK ASO and OSR1 ASO to the tFNA and synthesizing via the one‐pot annealing method. In primary ChP epithelial cells, TSOs revealed a remarkable ability to reduce the expression of SPAK and OSR1, and also their downstream ion channel proteins, which play pivotal roles in CSF secretion. Besides, TSOs effectively blocked the transition of macrophages from M0 to M1 phenotype by inhibiting the MAPK signaling pathway and inhibited proinflammatory cytokines such as TNF‐α, IL‐1β, and CCL2. In the rat hydrocephalus model, TSOs effectively decreased the CSF secretion rate and CSF volume. Notably, TSOs efficiently downregulated the in vivo expression of SPAK, OSR1, NKCC1, and pNKCC1, and also inhibited macrophage‐mediated choroid plexus inflammation. Our present work established a novel and promising treatment approach for preventing hydrocephalus after IVH and infection and may have critical clinical applications in the future.

## 4 Experimental Section

### Synthesis of TSOs

Firstly, SPAK ASO was attached to the 3ʹ end of S2 to form a new sequence. OSR1 ASO was then attached to the 3ʹ end of S4 (see Table S1, Supporting Information). Then these four ssDNA were mixed in TM buffer (containing 10 mM Tris‐HCl and 50 mM MgCl_2_) and synthesized to TSOs at 95 °C for 10 min and 4 °C for 20 min.

### Characterization of TSOs

8% polyacrylamide gel electrophoresis (PAGE) was used to verify the successful formation of TSOs. DLS (Nano ZS; Malvern Panalytical, England) was used to calculate the average zeta potentials and sizes. Atomic force microscopy (AFM) and transmission electron microscopy (TEM) images were acquired using an SPM‐9700 instrument (Shimadzu, Kyoto, Japan) and a TEM at an accelerating voltage of 200 kV (JEM‐2100F, JEOL, Japan).

### Cell Culture and Treatment

LPS (1 µg ml^−1^) was used to simulate the inflammation in vitro experiments. Treatment groups were LPS (1 µg ml^−1^) co‐cultured with TSOs. The primary ChP epithelial cells from rats were cultured as previously described.^[^ ^61^ ^]^ The rat macrophage NR8383 cells were purchased from the National Collection of Authenticated Cell Cultures (Beijing, China). Primary rat myeloid cells were isolated from the SD rat tibia and then differentiated into BM‐derived macrophages with M‐CSF (20 ng ml^−1^). For NR8383 cells, they were treated with TSOs or LPS incubation for 24 h, then the different treated cells were collected. The cells were placed in a 5% CO2 incubator at 37 °C in MEM containing 1% penicillin‐streptomycin and 10% FBS.

### Cell Viability

The primary ChP epithelial and NR8383 cells were cultured in a 96‐well plate and then treated with different concentrations of TSOs (50, 100, 200, 250, and 300 nM). After incubation for 24 h, 10% CCK‐8 reagent (Vazyme, Nanjing, China) was added and incubated for at least 1 h at 37 °C.

### Cellular Uptake of TSOs

To explore whether TSOs could be absorbed by primary ChP epithelial and NR8383 cells. The cells were cultured in a 24‐well plate and then co‐incubated with Cy5‐labeled TSOs(250nM) at different times. Finally, the cells were stained with fluorescein isothiocyanate (FITC) and DAPI. Then, a confocal laser microscope (Nikon, Japan) was used to observe the TSO uptake images in cells. For flow cytometry, the cells were seeded in a 6‐well plate and treated with Cy5‐labeled TSOs (250nM). Finally, the relative fluorescence intensity of Cy5 was detected using a flow cytometer (Beckman, Cytoflex).

### Immunofluorescence Staining Assays

The cells were fixed with 4% paraformaldehyde after different treatments, permeabilized with 0.2% TritonX‐100, and blocked with 1% BSA. The following diluted primary antibodies were incubated at 4 °C overnight: SPAK (1:100, ab128894, Abcam), OSR1 (1:100, 15611‐1‐AP, Proteintech), NKCC1 (1:100, ab303518, Abcam), pNKCC1 (1:100, ABS1004, Sigma), CD86 (1:20, sc‐28347, Santa), CD68 (1:100, 28058‐1‐AP, Proteintech), iNOS (1:250, ab178945, Abcam), IBA‐1 (1:100, 10904‐1‐AP, Proteintech), TNF‐α (1:500, ab183218, Abcam), IL‐1β (1:100, ab254360, Abcam), and CCL2(1:100, ab7202, Abcam). Then, they were incubated with the secondary antibody (Abcam) at ambient temperature for 1 h. The cytoskeletons were stained with phalloidin (Beyotime, Shanghai, China) and the nucleus was stained with DAPI. Subsequently, the fluorescence was observed using a confocal laser microscope (Nikon C2, Japan).

For the brain sections, the SD rats were anesthetized with 3% isoflurane and transcardially perfused with ice‐cold saline and 4% paraformaldehyde (PFA). Rat brains were dissected and kept in formalin for 24 h, followed by transferring to a 30% sucrose solution for cryoprotection. Brains were cryosectioned (10‐µm slices, coronal), and incubated with a blocking solution at room temperature) followed by incubation with primary antibodies at 4 °C overnight: SPAK (1:100, ab128894, Abcam), OSR1 (1:100, 15611‐1‐AP, Proteintech), NKCC1 (1:100, ab303518, Abcam), pNKCC1 (1:100, ABS1004, Sigma), IL‐1β (1:100, ab254360, Abcam), IBA‐1 (1:100, 10904‐1‐AP, Proteintech), TNF‐α, ZO‐1 (1:100, ab251568, Abcam), and CD68(1:100, 28058‐1‐AP, Proteintech). On the following day, the brain slices were washed three times in PBS. Then, they were incubated with the respective secondary antibodies (Abcam). The nuclei were stained with DAPI after washing three times with PBS.

### Western Blotting

After different treatments, the cells were lysed in RIPA buffer supplemented with both protease and phosphatase inhibitors. Subsequently, the protein concentration was measured using a BCA kit (Vazyme, China) kit. The samples were separated using 10% SDS‐PAGE and then were transferred onto polyvinylidene fluoride (PVDF) membranes. The membranes were blocked with 5% bovine serum albumin (BSA) for 1 h and then incubated with the following primary antibodies at 4 °C overnight: (SPAK (1:1000, ab128894, Abcam), OSR1(1:1000,15611‐1‐AP, Proteintech), NKCC1 (1:1000, ab303518, Abcam), pNKCC1 (1:1000, ABS1004, Sigma), iNOS (1:1000, ab178945, Abcam), pNF‐κB (1:1000, 3033, CST), TNF‐a (1:1000, 60291‐1‐Ig, Proteintech), CD206 (1:1000, 18704‐1‐AP, Proteintech), IL‐10 (1:1000, ab9969, Abcam), p38 (1:1000, 8690, CST), pP38(1:1000, 4511, CST), ERK1/2 (1:1000, 4695, CST), and pERK1/2 (1:1000, 4370, CST). Subsequently, the membranes were incubated with secondary antibodies for 1 h at room temperature. Ultimately, the chemiluminescence method was employed to assess the protein expression.

### Establishment of a Model of Post‐Hemorrhagic Hydrocephalus

The surgical procedures for intraventricular hemorrhage (IVH) models were developed based on previously published methods. After successful anesthesia and appropriate preparation of the surgical site. Male rats weighing 250 g were positioned in a stereotaxic frame, and then a cranial burr hole (1 mm) was drilled (coordinates: 0.6 mm posterior and 1.6 mm lateral to bregma). Subsequently, the right femoral artery was catheterized to collect the blood sample. With the help of a stereotaxic apparatus, a 29‐gauge needle was inserted into the brain at a depth of 4.5 mm from the dura mater. Finally, 200 µl of nonheparinized arterial blood was infused at a rate of 14 µl min^−1^. Then, the needle was held in place for an additional 10 min to prevent backflow of blood. The rats were randomized to Shame, IVH+Saline, and IVH+TSOs groups. For the treatment groups, following the injection of blood, a ventricular infusion tube was placed in this hole and fixed. TSO‐treated rats received TSOs (10 µl, 250 nM) every 12 h starting at the IVH and continuing until 48 h after IVH. The IVH group received saline injection through the tube. After the 48‐h treatment period, the infusion tubes were removed, and the rats were subjected to cranial MRI examinations.

### 7‐T Magnetic Resonance Imaging Scan and Ventricular Volume Analysis

Rats were anesthetized with 2% isoflurane throughout the MRI examination. The MRI scans were conducted using a 7.0‐T Varian MR scanner (Bruker, USA), employing a T2* gradient‐echo sequence and a T2 fast spin‐echo sequence. To ensure stability during the scans, the rats were fixed within a frame. Ventricular images were acquired precisely 48 h post‐hemorrhage. The 3D reconstruction of the ventricular system was achieved using the 3D Slicer software tool.

### Rat Choroid Plexus Epithelium Harvesting and Primary Cultures of CPECs

Firstly, the rats were euthanized, and their brains were perfused with ice‐cold PBS through the transcardial method. Subsequently, both the lateral and fourth ventricle choroid plexus were meticulously isolated under a microscope and transferred to 1.5 ml tubes. The choroid plexus (ChP) tissues were then finely dissected using a surgical knife and subjected to trypsin digestion. To ensure cell dissociation, the tissue was triturated multiple times using a 200 µl pipette. Following centrifugation, the cell sediment was resuspended in CPEC media supplemented with 10 000 units per ml Pen‐Strep, 10% FBS, and 10 ng ml^−1^ human EGF in DMEM. These cells were subsequently plated onto culture dishes and maintained at 37 °C with 5% CO2 for a period of 7 days.

### CSF Cytokines Collection

In different groups of 48 h after surgery for the PHH model. The anesthetized rats were securely placed in a stereotactic apparatus with their heads carefully positioned perpendicular to the ground, facilitating the exposure of the atlanto‐occipital ligament. Subsequently, a syringe was carefully inserted into the cisterna magna, and ≈100 µl of cerebrospinal fluid (CSF) was meticulously collected into a 1.5 ml tube. To eliminate tissue debris, the collected CSF underwent centrifugation at 10 000× g for 5 min before being stored at −80 °C for further analysis.

### Quantitative PCR Analysis

Total RNA from the samples was extracted using TRIzol reagent (Thermo Fisher Scientific). The cDNA was prepared using a cDNA reverse transcription kit (Vazyme, Nanjing, China). The quantitative PCR was used to amplify the target mRNAs. The primer sets of the targeted genes are listed in Table S2, Supporting Information. The expression of targeted genes was normalized to GAPDH and evaluated.

### Statistical Analysis

Statistical analysis was performed using GraphPad Prism 8.0 (GraphPad, Boston, MA, USA). The Student's *t*‐test and one‐way ANOVA were used to analyze the significant difference between the samples in the experiments. *p < 0.05, **p < 0.01, ***p < 0.001, and ****p < 0.0001 were used as thresholds for statistical significance.

## Conflict of Interest

The authors declare no competing interests.

## Author Contributions

Q.W., H.X., and Y.L. conceived and designed this research. Q.W. performed most of the work. The authors gratefully acknowledge the RNA‐seq analysis assistance from J.C., synthesis and characteristic of TSOs from W.M. and Y.Z., rat models and tissue slices from Y.L. and H.Z., DLS and MRI images capture from F.L. and X.L., data collection and analysis from Y.J. and L.M. Manuscript was written by Q.W. and revised by X.H. and Y.L. All authors discussed the results and contributed to the final manuscript.

## Supporting information

Supporting Information

## Data Availability

The data that support the findings of this study are available from the corresponding author upon reasonable request.
