# Supplementary material for: Modulation of Cerebrospinal Fluid Dysregulation via a SPAK and OSR1 Targeted Framework Nucleic Acid in Hydrocephalus
Source: Adv Sci (Weinh). 2024 Feb 14;11(17):2306622. doi: 10.1002/advs.202306622 (PMC11077654; doi:10.1002/advs.202306622)
Supplement: Supplementary file 1 — Supporting Information [file ADVS-11-2306622-s001.pdf]

ADVANCED SCIENCE
Open Access

Supporting Information

for *Adv. Sci.*, DOI 10.1002/advs.202306622

Modulation of Cerebrospinal Fluid Dysregulation via a SPAK and OSR1 Targeted Framework Nucleic Acid in Hydrocephalus

Qiguang Wang, Jian Cheng, Fei Liu, Jianwei Zhu, Yue Li, Yuxuan Zhao, Xiang Li, Huan Zhang, Yan Ju, Lu Ma, Xuhui Hui and Yunfeng Lin**

Supporting Information

Modulation of cerebrospinal fluid dysregulation via a SPAK and OSR1 targeted Framework Nucleic Acid in hydrocephalus

Qiguang Wang[1], Jian Cheng[1], Fei Liu[3], Jianwei Zhu[4], Yue Li[5], Yuxuan Zhao[2], Xiang Li[1], Huan Zhang[1], Yan Ju[1], Lu Ma[1], Xuhui Hui[1], Yunfeng Lin[2,6,7]

Affiliations:

1.Department of Neurosurgery, West China Hospital, Sichuan University, Chengdu, Sichuan, 610041, China

2.State Key Laboratory of Oral Diseases, National Center for Stomatology, National Clinical Research Center for Oral Diseases, West China Hospital of Stomatology, Sichuan University, Chengdu 61004, P. R. China;

3.Institutes for Systems Genetics, Frontiers Science Center for Disease-Related Molecular Network, West China Hospital, Sichuan University, Chengdu, China.

4.Department of Neurosurgery, Sichuan Provincial People's Hospital, University of Electronic Science and Technology of China, Chengdu.

5.Core facilities, West China Hospital, Sichuan University, Chengdu, Sichuan, 610041, China.

6. Sichuan Provincial Engineering Research Center of Oral Biomaterials, Chengdu, Sichuan 610041, China

7. National Center for Translational Medicine, Shanghai Jiao Tong University, Shanghai 200240, China

Corresponding Author:

Yunfeng Lin

State Key Laboratory of Oral Diseases National Clinical Research Center for Oral Diseases, West China Hospital of Stomatology, Sichuan University, Chengdu 61004, P. R. China; Sichuan Provincial Engineering Research Center of Oral Biomaterials, Chengdu, Sichuan 610041, China; orcid.org/0000-0003-1224-6561;

Email: yunfenglin@scu.edu.cn

Xuhui Hui

Department of Neurosurgery, West China Hospital, Sichuan University, Chengdu, Sichuan, 610041, China

Email: huixuhui@126.com

Experimental Section:

1. Stability analysis of TSOs

1.1 Storage stability

The samples were kept within a refrigerator at a temperature of 4°C, and on a daily basis over the course of a week, they were retrieved to undergo PAGE gel electrophoresis testing.

1.2 Serum Stability

Different concentrations of FBS (5%, 10%, 15%, 20%, 30%, 40%) were added to TSOs and placed in an incubator at 37°C for 24 h before the PAGE test.

1.3 pH and salt solutions stability

To analyze the pH stability, TSOs were incubated in TM buffer with different pH values at 37 ℃ for 2h, and analyzed by PAGE. In order to analyze the salt solution stability, TSOs was incubated in 10% NaCl and $MgCl_2$ solution at 37℃ for 2h, and then analyzed by PAGE.

2. Transwell assay:

The cell density was adjusted to an optimal level using a serum-free high-sugar DMEM medium, and subsequent inoculation took place within a Transwell chamber. Following cell adhesion to the chamber walls, distinct compounds were introduced based on the aforementioned groupings, and a 24-hour cultivation ensued. After sample collection, they were fixed with 4% paraformaldehyde fixative solution for 15 min. After PBS cleaning for twice, the transwell chambers were stained with crystal violet for at least

20 minutes. Then the cells in the inner membrane of the upper chamber was wiped out. Subsequently, they were immediately placed under a positive fluorescence microscope for observation and images acquisition.

3. Hematoxylin-Eosin staining:

The paraffin-embedded samples were prepared into 3 μm thick paraffin sections. Then the tissues are first deparaffinized and rehydrated, followed by immersion in hematoxylin dye for 10 min. Subsequently rinsed with water, and then introduced into a saturated lithium carbonate solution for 30 seconds. Then the slices were washed again and subjected to eosin staining for a duration of 2 minutes. A gradient alcohol immersion facilitated dehydration and transparency after cleansed with water. Finally, Permount TM Mounting Medium was added for storage.

4. Measurement of CSF production rates:

The method for measuring the rate of CSF secretion was described by Karimy et al[9]. Briefly, the rats were anesthetized and positioned on a stereotactic apparatus, then a cranial burr hole(1mm) was drilled over the left ventricle (coordinates:0.6 mm posterior and 1.6mm lateral to bregma). A ventricular infusion tube, as previously mentioned, was then inserted and fixed on the burr hole, with a depth of 4.5 mm. Next, the rat's head was gently rotated by 90° using the ear bars, positioning it with the nose pointing downward. Suboccipital muscles were dissected to expose the atlantooccipital ligament. A 29-gauge needle was punctured through the ligament and advanced 5mm through the foramen of Magendie to the fourth ventricle. To create a closed CSF circulation in the lateral ventricles, we then used sterile molecular-grade mineral oil (100 μl; Sigma-Aldrich) to occlude the aqueduct Sylvius, thereby preventing CSF flow from the third ventricle to the fourth ventricles. Once the rat remained in the same position, a straight PE tube (OD:0.5mm, ID:1mm, length:20 cm) was connected to the fix tube. The PE tube should be flush with the fixed tube and should be perpendicular to the rat's head. The volume of CSF collected over a given time (40 min) was calculated using the formula V (mm3) = $\pi \cdot r^2 \cdot d$, where "r" represents the radius of the PE tube and "d"

indicates the distance that CSF traveled within the tube. Consequently, the rate of CSF formation(ul/min) was the calculated.

5. Transcriptome sequencing analysis

The NR8383 cells were cultured in 12-well plate and divided into two groups. The first group received stimulation with LPS(1ug/ml) (n=3), while the second group was exposed to both LPS (1 μg/ml) and TSOs (250 nM) (n=3). After 24 hours, the cells were harvested, and total RNA was isolated using the TRIzol reagent (Invitrogen, USA). Subsequently, quantification and purity assessment of the RNA were performed using a NanoDrop ND-1000 instrument (NanoDrop, USA). After detecting the RNA integrity, the RNA samples underwent processing at Novogene Bioinformatics Technology Co., Ltd (Tianjin, China). RNA-Seq libraries were generated utilizing the Illumina standard mRNA-seq library preparation kit and sequenced by the Illumina NovaSeq 6000. The thresholds of significantly different expression were $p < 0.05$ and $|\log2foldchange| > 0.6$. The GO and KEGG databases were used to explore the functions and biological pathways in which the differentially expressed genes were involved.

Results and Discussion:

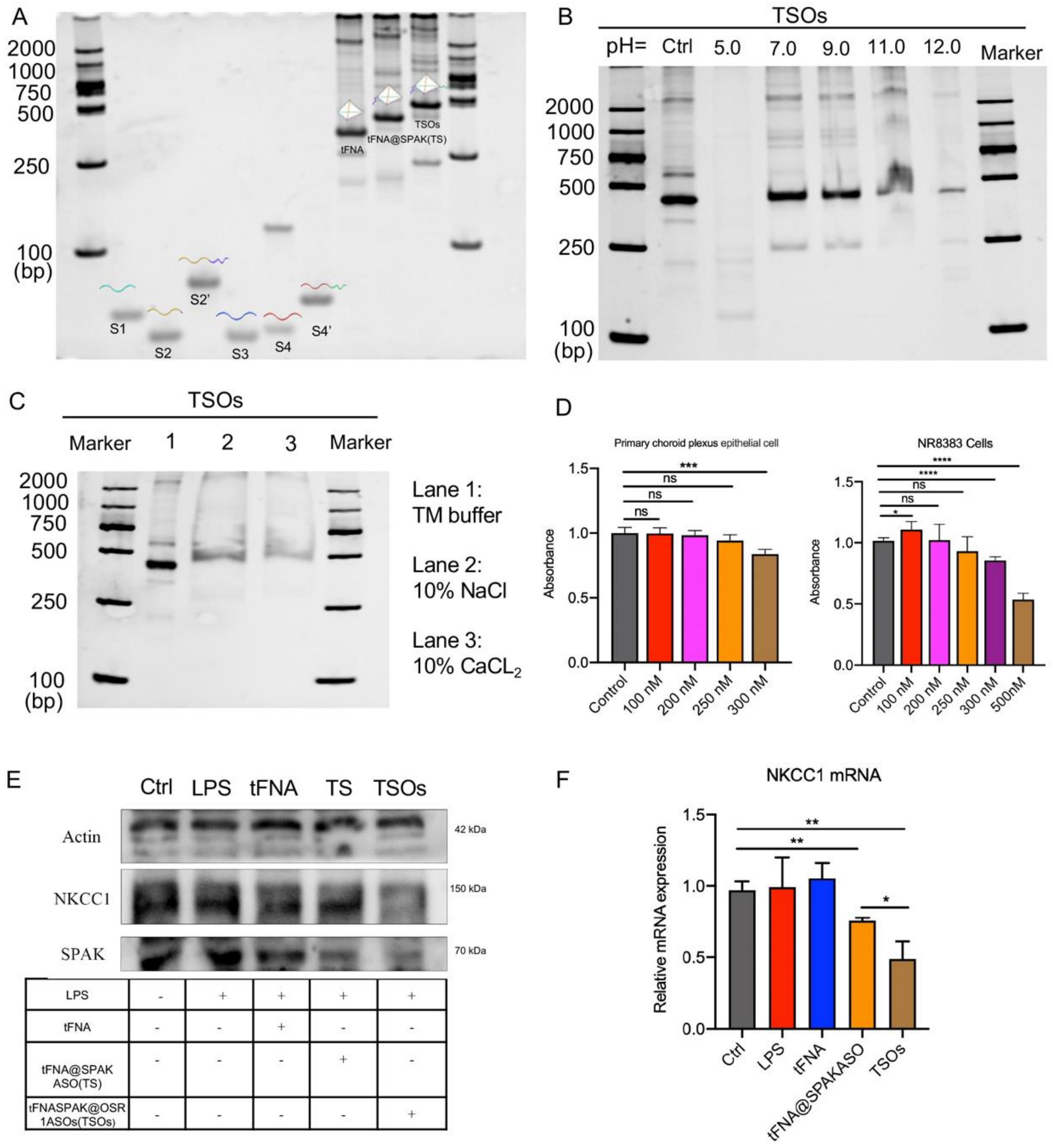

Supporting Information Figure S1: Evaluation of successful synthesis of TSOs, effect on cells viability and the knockdown efficiency. (A) 8% PAGE verified the successful synthesis of the modified S2', S4' and TSOs. (B) PAGE showing the stability of TSOs in TM buffer with different pH values. (C)PAGE showing the stability of TSOs in different salt solutions. (D) The biosafety of TSOs in primary choroid plexus epithelial cells and rat macrophages NR8383. Western Blotting(E) and QPCR (F) show that targeting both the SPAK and OSR1 yields superior efficiency compared to solely targeting SPAK.

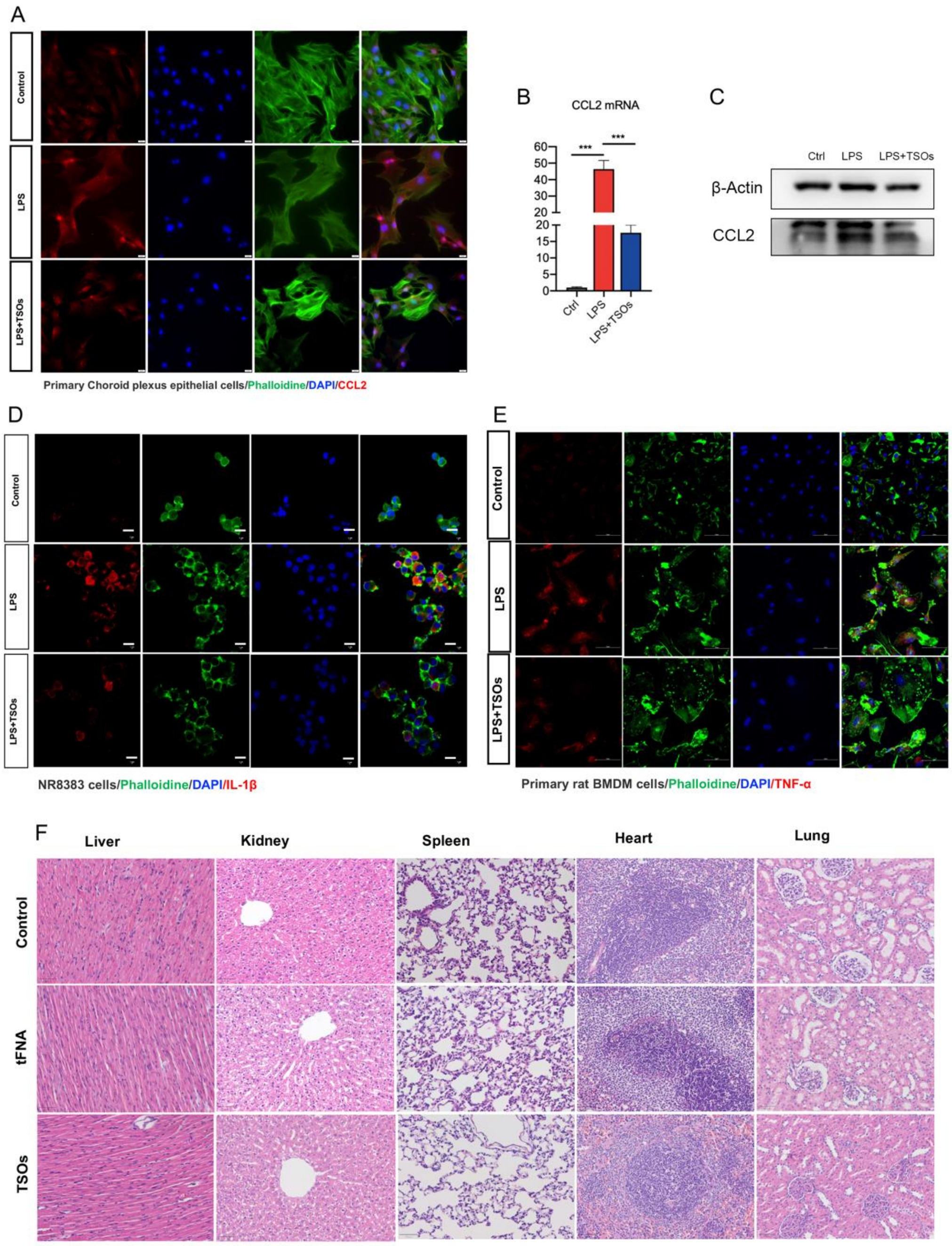

Supporting Information Figure S2: CCL2 expression in Primary Choroid plexus epithelial cells and TNF-α expression in primary rat BMDM cells after TSOs treatment, and evaluation of TSOs biosafety in vivo. The IF staining(A), QPCR(B) and Western Blotting(C) depict that TSOs inhibited the expression level of CCL2 in primary choroid plexus epithelial cells after LPS stimulation. (D) The expression of IL-1β was assessed by IF staining after TSOs treatment in NR8383 cells. (E) The expression of TNF-α was assessed by IF staining after TSOs treatment in primary rat BM-derived macrophages. (F) H&E staining showing no obvious histological abnormalities in major organs (heart, liver, kidney, lung, spleen) after either tFNA and TSOs treatment.

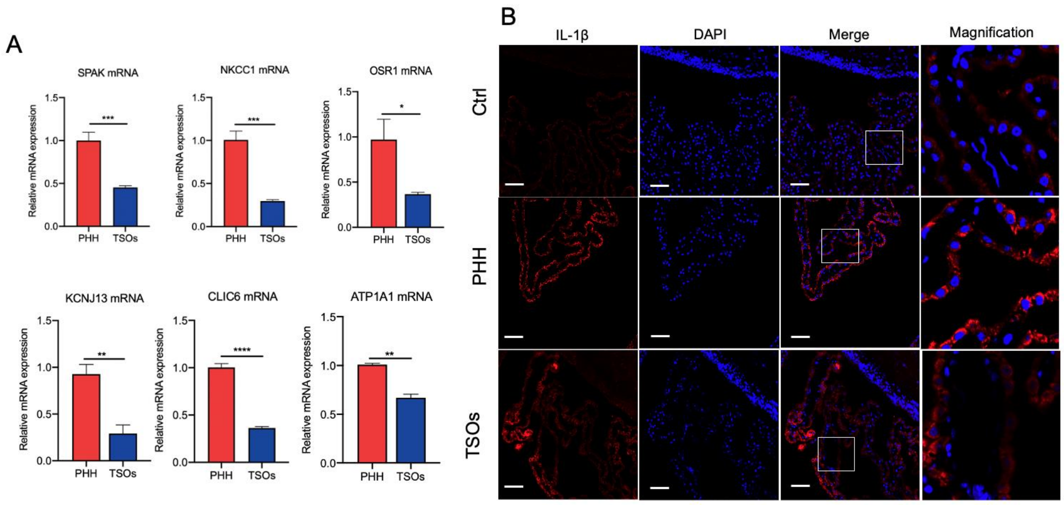

Supporting Information Figure S3: The knockdown efficiency of TSOs on the mRNA expression of SPAK, OSR1 and their downstream genes in vivo, and the expression of IL-1β in choroid plexus after TSOs treatment. (A) TSOs downregulated the expression of SPAK, OSR1, NKCC1, ATP1A1, KCNJ13 and CLIC6 at mRNA level in vivo. (B) IF staining of IL-1β in choroid plexus in rat PHH treated with TSOs.

Table S1. Base sequence of single stranded oligonucleotides

ssDNA	Base sequence (5’→3‘)
S1	ATTTATCACCCGCCATAGTAGACGTATCACCAGGCAGTTGAGACGAACATTCCTAAGTCTGAA
S2	ACATGCGAGGGTCCAATACCGACGATTACAGCTTGCTACACGATTCAGACTTAGGAATGTTCG
S2'(modified S2)	ACATGCGAGGGTCCAATACCGACGATTACAGCTTGCTACACGATTCAGACTTAGGAATGTTCG TTTTTGGCTCCGCCATGATGCTGC
S3	ACTACTATGGCGGGTGATAAAACGTGTAGCAAGCTGTAATCGACGGGAAGAGCATGCCCATCC
S4	ACGGTATTGGACCCTCGCATGACTCAACTGCCTGGTGATACGAGGATGGGCATGCTCTTCCCG
S4'(modified S4)	ACGGTATTGGACCCTCGCATGACTCAACTGCCTGGTGATACGAGGATGGGCATGCTCTTCCCG TTTTCCTCGGACATGACGGCTGC

Table S2. Primer Sequence of Gene designed for Q-PCR.

**mRNA**		**Primer pairs**
CLIC6	FP	CTGCCCATTCTCACAGCGTCTC
	RP	TTCAGGTCCACCGTTGTCACATTG
iNOS	FP	AAGAGACGCACAGGCAGAGG
	RP	AGCAGGCACACGCAATGATG
GM-CSF	FP	GACATGCGTGCTCTGGAGAAC
	RP	GCCTCTGGATGGAGAACTCATTAG
KCNJ13	FP	GTCCCGCCTGAAAACCACACTATC
	RP	TGGTGCCATAGCCGATTGTAAGTTG
ATP1A1	FP	GTCTGCTGTGGCTCCGTGATG
	RP	TGCTTAGGCTCCGATGCGTTTG
GAPDH	FP	ACGGCAAGTTCAACGGCACAG
	RP	CGACATACTCAGCACCAGCATCAC
CD86	FP	GCTGTCTCTTTCTGCTGGTCGTC
	RP	CTCACAAGTCTTTCTGCTGGGTCTG
OSR1	FP	CGCCTGCCTTCCAAGACCAAG
	RP	GTGCGTCCGCTCGTGGATAAG

NKCC1	FP	GGGTCAGGCTGGAATAGGTCTCTC
	RP	ATGCTCCTCCTCCTCTCACGAATC
SPAK	FP	TTGCTGACACGAACACCAGACATAG
	RP	CCAGTCGCCATCTTCAGTCTTGTG
IL-1α	FP	AAGCCTGTGTTGCTGAAGGAGATTC
	RP	CTCTGGGAAAGCTGCGGATGTG
TNF-α	FP	ATGGGCTCCCTCTCATCAGTTCC
	RP	CCTCCGCTTGGTGGTTTGCTAC
IL-1β	FP	AATCTCACAGCAGCATCTCGACAAG
	RP	TCCACGGGCAAGACATAGGTAGC
CCL2	FP	CTCACCTGCTGCTACTCATTCACTG
	RP	CTTCTTTGGGACACCTGCTGCTG
IL-17	FP	CCTGATGCTGTTGCTGCTACTG
	RP	GGACACACTGAACTTTGAGGGATG
CD40	FP	CTTGTTGACAGCGGTCCATCTAGG
	RP	CACGGTTGGCATTGGGTCTTCTC
